# Corrosive Sublimate in Dysentery

**Published:** 1878-04-20

**Authors:** Charles H. Hall

**Affiliations:** Macon, Ga.


### CORROSIVE SUBLIMATE IN DYSENTERY.

By Charles H. Hall, M.D., of Macon, Ga.

A married woman, of twenty-eight, applied to me through her husband. For two months she had been daily having from ten to twenty straining, painful operations, fæces sometimes natural, yet afterward mucus and blood; and again fæces soft, mucus and blood intermixed. He represented that she had no fever; may possibly have had some in the beginning; she was weak; had no appetite and suffered continual pain. Had been treated by a physician with castor oil, frequently repeated as a purgative, opium, etc. I gave him one-half grain of corrosive sublimate, dissolved in eight ounces of water, directing that she should take four teaspoonsfuls of this each day. He reported in one week that his wife was very much better, but still had four or five actions each day, and very much of the same character. I continued the mercurial, same dose, and directed five grains of sulphate of copper in a pint of water, to be used twice daily by enema. Reported in a week that his wife was well.

A negro man of fifty came to me first of January, 1877. For six months he had been troubled each day with teasing desire to stool, and would pass mucus and blood at each effort; no fever; considerable emaciation. Tongue was so coated with tobacco that I could gain no information from its appearance. There were no piles; neither had been. He had taken castor oil, opium and other medicines as prescribed. My diagnosis was catarrh of rectum. I gave him half gr. corrosive sublimate in a pint of water, directing him to take a teaspoonful every two hours. He reported in a week, saying he was much better than he had been for months. Continued treatment; met him accidentally one month afterward; he said he was entirely well and working every day.

November, 1876. A lady of twenty-six years. For eighteen months she had suffered with frequent discharges from her bowels. For days she would have small, frequent and intensely painful discharges of mucus and blood. Suddenly these would cease, and she would have large, very thin and offensive actions, and these very frequent. She was greatly emaciated, her tongue reddish, very smooth, something like the surface of a glass. Her appetite was variable, sometimes morbid, and then again, having a loathing of all food. Her abdomen was tender and flat. She was up, and attempting to keep house, but very feeble. She had been under various treatments, lay and professional; was convinced that no good would result from any treatment, especially averse to "bad-tasting medicine." Diagnosing a chronic catarrhal condition of whole alimentary mucus membrane, I prescribed one grain of corrosive sublimate to a pint of water, teaspoonful every two hours; five grains of salicin three times a day. To have, per anum, an injection of sulphate of copper (five grains to a pint of water.) Salicin was discontinued after one week, and the corrosive sublimate continued for three weeks longer. In January, two months after my first visit, she had gained flesh wonderfully. Her actions were free from blood and mucus, and had been for four weeks. In twenty-four hours she would have from two to five rather loose actions. Abdomen was no longer tender. She was very much stronger, and very hopeful of final cure. Bismuth and tonic were prescribed. First of March she reported herself well.

A girl of eleven years. For months she had had an affection of the bowels; was very pale; had lost much flesh; appetite variable and capricious. She would have a natural-looking action, but accompanied with great pain, and around the fæces mucus and blood. This probably once or twice in twenty-four hours, but all through the day and night, as much as fifteen or twenty times, she would have small discharges of nothing but mucus and blood, with great straining and pain. She had been treated by many doctors. I examined her rectum, but could discover no fissure, ulcer, or piles. Diagnosing a chronic catarrh of rectum, prescribed small doses of mercury and chalk; and as her digestion was imperfect, pepsin, ten grains with each meal. She soon evidenced improvement, and in three months was dismissed perfectly well.

A lady, about twenty-five, had been three weeks sick with rheumatism; was taken with an intercurrent dysenterry; stools very frequent, slimy and bloody; great tenderness. I found her taking laudanum very freely. She was the patient of another physician; he being called out of town, I was called in. I stopped the laudanum and the rheumatic remedies; gave her one hundredth of a grain of corrosive sublimate every two hours. In twelve hours she was greatly relieved, and in forty-eight hours had no further trouble.

A quadroon woman, of twenty or twenty-five years, had been sick three days, discharging from her bowels blood and mucus, with a great deal of tenesmus, considerable tenderness over the whole abdomen; high fever. Prescribed castor oil; quinine, as an antipyretic, twenty grains in two doses. Turpentine stupes over the abdomen; laudanum to be given as soon as oil acted. Next day my patient was no better in any particular; oil had acted, laudanum, etc., been given.

Continued quinine (eighteen grains in two doses ;) ordered one-half grain of corrosive sublimate in half a pint of water, teaspoonful every two hours. Next day fever was not quite so high, dysentery possibly slightly better, discharges not so frequent and tenesmus not so distressing. Continued quinine and corrosive sublimate. Fourth day—Temperature normal; tenderness over the abdomen greatly improved; reported only two actions since last visit, much more fœcal in character. Stopped quinine, continued mercury. Patient had no further trouble.

These cases are taken from my case-book, to illustrate the efficacy of "small and frequently repeated doses" of mercury in this disease. There cannot be any doubt of the success, in the great majority of cases, of this method of treatment. I could furnish records of many more successful cases, and a few unsuccessful ones, treated in this manner. My success so far has been very gratifying, greatly preponderating. Ringer, who advises it in his book, deserves no credit for it except for popularizing it. Any one curious on the subject of his small doses, not only in this disease, but in almost every other one of his recommendations, has only to refer to homœpathic works and find that he has plagiarized. Take up any one of their works, even the domestic manuals of twenty-five years ago, and you will find corrosive sublimate put at the head of the list of remedies in dysentery. Although a regular physician of the strictest sect, I believe we should give credit even to irregulars where they deserve it.—*Med. and Surg. Rep.*

